# m6A and m5C modifications as the gears: *CmoCK1* mRNA travels to promote chilling tolerance

**DOI:** 10.1093/plphys/kiae572

**Published:** 2024-10-30

**Authors:** Yee-Shan Ku

**Affiliations:** Plant Physiology, American Society of Plant Biologists; School of Life Sciences and Centre for Soybean Research of the State Key Laboratory of Agrobiotechnology, The Chinese University of Hong Kong, Hong Kong SAR, China

Long-distance signaling in plants allows the transmission of stimuli from sensing organs to target organs. Such a transmission enables precise and timely responses to stresses. The signal transmitters include various molecules such as peptides, small proteins, metabolites, and RNAs (Takahashi and Shinozaki 2019). For example, mobile mRNA transported between shoot and root mediates the chilling tolerance of heterografted watermelon plants (Wang et al. 2020). Proposed factors underlying the mRNA mobility include their abundance and other features such as TLS (tRNA-like sequence) motif and m5C (5-methylcytosine) RNA modification (Calderwood et al. 2016; Zhang et al. 2016; Kehr et al. 2022). However, the association between these factors and the mobility has remained inconclusive.

In this issue of *Plant Physiology*, Li et al. report the regulated mobility of pumpkin *CHOLINE KINASE 1* (*CmoCK1*) mRNA by m5C and m6A (6-methyladenine) modifications under chilling stress (Li et al. 2024). The authors first tried to identify possible common features regulating mRNA mobility in different species. They analyzed the length and TLS motifs of homologous mobile mRNAs existing in different plants, including Arabidopsis, *Vitis vinifera* (grapevine), *Cucumis sativus* (cucumber), *Cucurbitea moschata* (pumpkin), cucumber/*Cucumis lanatus* (watermelon) heterograft, and Arabidopsis/*Nicotiana benthamiana* heterograft (Liu et al. 2020; Li et al. 2024). However, both the length and TLS did not show correlations with the mobility. Therefore, it appeared that there are other unidentified regulators.

*AtCK1* (*CHOLINE KINASE 1*) is a well-characterized mobile mRNA in Arabidopsis (Zhang et al. 2016), and its orthologs have been reported in other plant species. In this study by Li et al., by searching through published datasets in different species, the authors found that pumpkin *CmoCK1* mRNA has both shoot-to-root and root-to-shoot mobility (Li et al. 2024). Interestingly, under chilling, the expression of *CmoCK1* was first induced in root and then in leaf. Before *CmoCK1* is induced in leaf, *CmoCK1* mRNA is able to move from root to leaf (Li et al. 2024). Using *CmoCK1*-*GUS* reporter fusion construct, the authors also validated the translation of the mobile mRNA to a functional protein in the destined tissue. To further validate the functions of *CmoCK1*, chilling tolerance tests were conducted using cucumber as the model. The overexpression of *CmoCK1-GFP* in cucumber promoted chilling tolerance, reduced O_2_ levels, decreased the relative electrolyte permeability, and induced cold responsive genes (Li et al. 2024). Moreover, when grafted to the wild-type cucumber shoot, cucumber roots overexpressing *CmoCK1* conferred chilling tolerance to the heterograft (Fig.).

**Figure.**
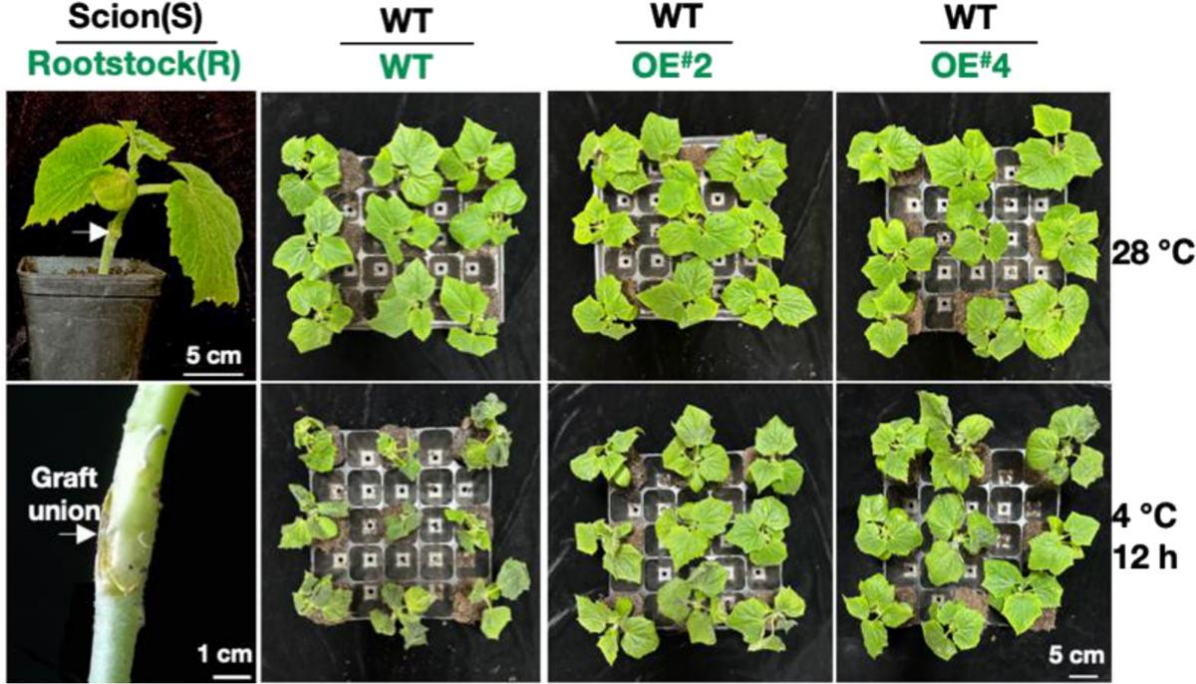
*CmoCK1* promotes chilling tolerance. Cucumber rootstocks overexpressing *CmoCK1* (OE^#^2, OE^#^4) were grafted with wild-type cucumber scion. Compared with the grafts composed of wild-type rootstock and wild-type scion, the grafts composed of *CmoCK1* overexpressing rootstock and wild-type scion were more tolerant to chilling at 4 °C. The figure is adapted from Li et al. 2024 (Li et al. 2024).

Interestingly, while *AtCK1* mRNA mobility has been shown to be regulated by TLS, *CmoCK1* mRNA lacks TLS. The authors therefore focused on other features, including m6A and m5C modifications. The fusion of m6A or m5C modification region to *YFP* rendered the mRNA mobile. While the deletion of m6A or m5C modification region decreased the mobility of *CmoCK1* mRNA, the deletion of both regions completely stopped the mobility (Li et al. 2024). Moreover, the deletion of the m6A modification region decreased the mRNA stability (Li et al. 2024). The results suggested that the m6A modification promotes mRNA mobility by maintaining its stability. Further experiments were done to validate the significance of m5C modification in mRNA mobility. *CsaTRM4B* encodes an m5C methyltransferase. Its co-transformation promoted the mobility of *CmoCK1* mRNA in *Nicotiana benthamiana*. However, when *CsaTRM4B* was replaced by the DNA methyltransferase inhibitor 5-azacytidine, the mobility of *CmoCK1* mRNA was decreased.

In summary, *CmoCK1* promotes chilling tolerance. Under chilling, its expression is first induced in root and then in leaf. Before it is induced in leaf, *CmoCK1* mRNA moves from root to leaf to promote the tolerance of the whole plant. Unlike its Arabidopsis homolog *AtCK1* mRNA, which has the mobility regulated by TLS, *CmoCK1* mRNA has its mobility regulated by m6A and m5C modifications. The study demonstrates species-specific mRNA mobility regulation and strengthens the emerging role of m6A in regulating mRNA mobility. Moreover, it suggests that m6A and m5C modifications contribute to the mobility by different mechanisms, adding one more dimension to the understanding of mRNA mobility.

## References

[kiae572-B1] Calderwood A , Kopriva S, Morris RJ. Transcript abundance explains mRNA mobility data in *Arabidopsis thaliana*. Plant Cell. 2016:28(3):610–615. 10.1105/tpc.15.00956 26952566 PMC4826013

[kiae572-B2] Kehr J , Morris RJ, Kragler F. Long-distance transported RNAs: from identity to function. Annu Rev Plant Biol. 2022:73:457–474. 10.1146/annurev-arplant-070121-033601 34910585

[kiae572-B3] Li X , Wang C, Liu W, Wang N, Zhang M, Chen Y, Xiang C, Gao L, Dong Y, Zhang W. The mobility of pumpkin *CHOLINE KINASE 1* mRNA is regulated by both m5C and m6A modification under chilling stress. Plant Physiol. 2024:kiae511. 10.1093/plphys/kiae511 39325727

[kiae572-B4] Liu W , Xiang C, Li X, Wang T, Lu X, Liu Z, Gao L, Zhang W. Identification of long-distance transmissible mRNA between scion and rootstock in cucurbit seedling heterografts. Int J Mol Sci. 2020:21(15):5253. 10.3390/ijms21155253 32722102 PMC7432352

[kiae572-B5] Takahashi F , Shinozaki K. Long-distance signaling in plant stress response. Curr Opin Plant Biol. 2019:47:106–111. 10.1016/j.pbi.2018.10.006 30445314

[kiae572-B6] Wang Y , Wang L, Xing N, Wu X, Wu X, Wang B, Lu Z, Xu P, Tao Y, Li G, et al A universal pipeline for mobile mRNA detection and insights into heterografting advantages under chilling stress. Hortic Res. 2020:7:13. 10.1038/s41438-019-0236-1 32025316 PMC6994652

[kiae572-B7] Zhang W , Thieme CJ, Kollwig G, Apelt F, Yang L, Winter N, Andresen N, Walther D, Kragler F. tRNA-related sequences trigger systemic mRNA transport in plants. Plant Cell. 2016:28(6):1237–1249. 10.1105/tpc.15.01056 27268430 PMC4944404

